# Data on the effect of geological and meteorological parameters on indoor radon and thoron level- case study: Kermanshah, Iran

**DOI:** 10.1016/j.dib.2018.04.122

**Published:** 2018-05-04

**Authors:** Meghdad Pirsaheb, Farid Najafi, Lida Hemati, Touba Khosravi, Hooshmand Sharafi

**Affiliations:** a Research Center for Environmental Determinants of Health, Kermanshah University of Medical Sciences, Kermanshah, Iran; b Student Research Committee, Kermanshah University of Medical Sciences, Kermanshah, Iran

**Keywords:** Indoor radon, Indoor thoron, Geology, Meteorological parameters, Kermanshah, Iran

## Abstract

The present study was aimed to evaluate the relationship between indoor radon and thoron concentrations, geological and meteorological parameters. The radon and thoron concentrations were determined in three hospitals in Kermanshah, the west part of Iran, using the RTM-1688-2 radon meter. Also, the type and porosity of the underlying soil and the meteorological parameters such as temperature, humidity, atmospheric pressure, rainfall and wind speed were studied and the obtained results analyzed using STATA-Ver.8. In this study the obtained radon concentration was furthered in buildings which constructed on the soil with clayey gravel and sand feature than the soil with clay characteristic and little pasty with a significant difference (*P* < 0.05). While the lower coefficient about 1.3 was obtained in measured the thoron concentration and a significant difference was not observed. So the soil porosity can extremely effect on the indoor radon amount. Among all studied meteorological parameters, temperature has been determined as the most important meteorological parameter, influence the indoor radon and thoron concentrations.

**Specifications table**

**Table:** 

Subject area	Environmental Science
More specific subject area	Earth and Planetary Sciences
Type of data	Tables and figures
How data was achieved	In present study, 102 samples were determined for radon and thoron concentration in three hospitals in Kermanshah, the west part of Iran, using the RTM-1688-2 radon meter. The type and porosity of the underlying soil and meteorological parameters such as temperature, humidity, atmospheric pressure, rainfall and wind speed were studied. Finally, the raw data was analyzed using STATA-Ver.8.
Data format	Raw, analyzed
Experimental factors	In each time of radon and thoron concentration measurement, the radon and thoron concentrations simultaneously were reported with 95% confidence interval by the radon meter after 150 min of continuous air suction.
Experimental features	All samples analysing were performed according to the standard method.
Data source location	Kermanshah, Iran
Data accessibility	Data are included in this article

**Value of the data**

• The quality of indoor air, such as air quality outside the building, is important for human health [1], [2], [3], [4], [5], [6], [7], [8]. The data of this study is to measure two dangerous indoor pollutants (Radon and Thoron) [9], [10].
• The data of present study is suitable for the effect of geological and meteorological parameters on the indoor radon and thoron level in the western region of Iran (Kermanshah).
• While dealing with indoor radon inhalation has attracted more attention, there were no further information about the amount of thoron in indoor air. The data of this study could partly fill the above mentioned information gap.
• The obtained data of this study confirm that the soil type under the building and temperature can significantly influence indoor radon concentration in buildings.

## 1 Data

**Fig. 1:**
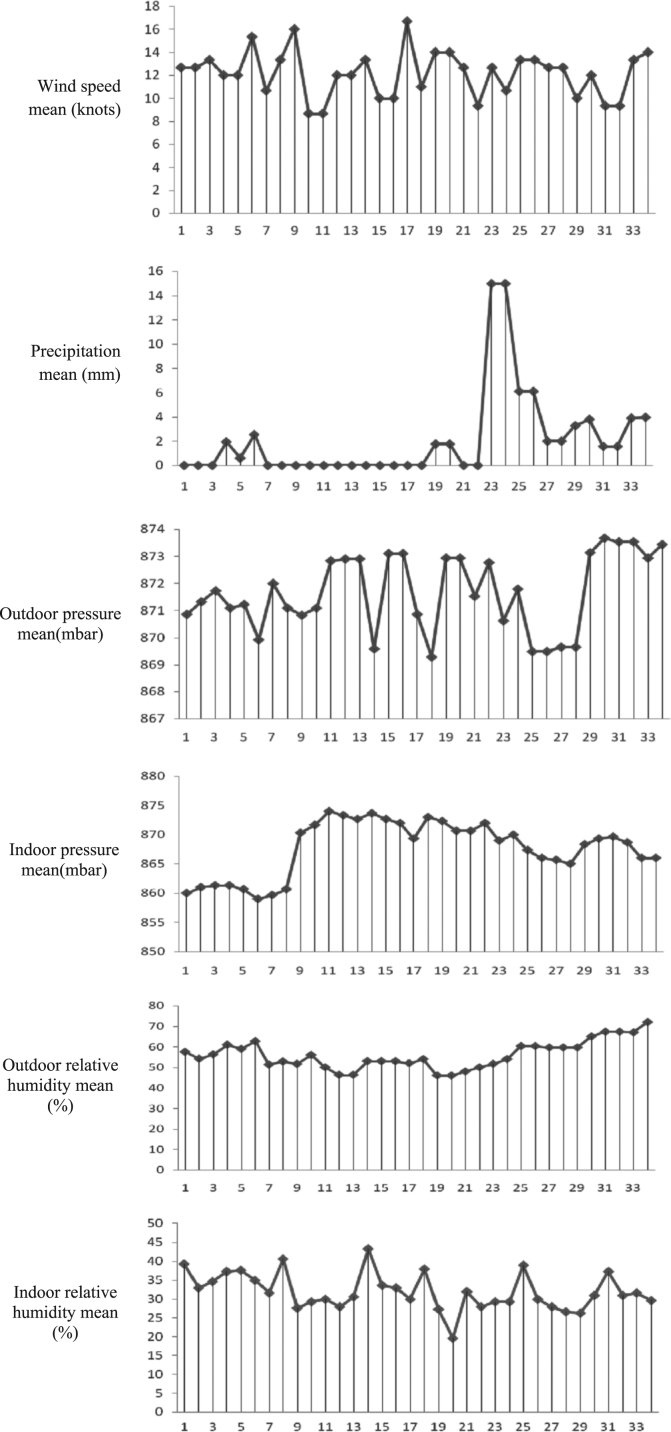

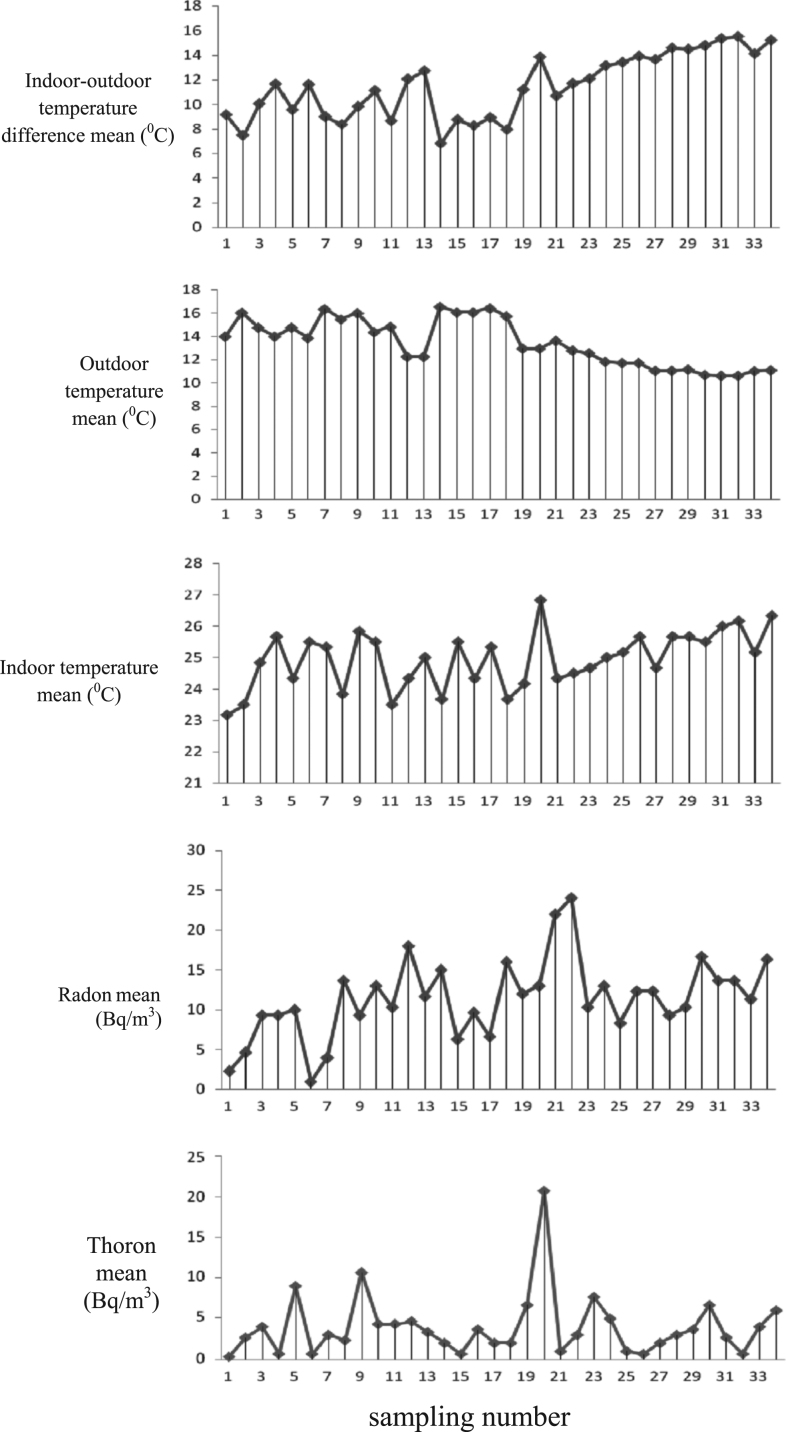
222Rn and 220Rn concentrations and meteorological variables.

Based on the results of present study, the average concentration of indoor radon and thoron in the studied hospitals were 11.44 ± 4.9 Bq/m^3^ and 4 ± 3.9 Bq/m^3^, respectively. Fig. 1 shows the variation of meteorological parameters, indoor radon and thoron concentrations. As shown in the Fig. 1, the wind speed or rainfall negatively effected on these gases, with increase the wind speed or rainfall the concentration of these gases decreases. Similarly, indoor and outdoor humidity negatively affected indoor radon and thoron concentrations. The indoor temperature seems to have a positive effects on the concentration of these gases, with increases it the concentrations of these gases increase. But with increased the outdoor temperature, the indoor concentrations of these gases decreased. This figure also showed that with increased the difference of indoor and outdoor temperature, the indoor radon and thoron concentrations declined. Because the outside air pressure was often in the range of 870–875 mbar and there are no variation, its influence on these gases is not obvious. While, the figure shows that the indoor radon concentration rises with increases the indoor air pressure. However, the radon concentration was in the highest and lowest level in Imam Reza (AS) (Table 1) and Imam Khomeini (RA) hospital (*P* = 0.003), respectively, but the multivariate regression model (Table 2) shows that the amount of radon in Imam Reza (AS) and Taleghani hospital were in the highest and lowest level, respectively, to compare with Imam Khomeini hospital. The similar form can be observed about the indoor thoron, although no significant differences were detected (*P* = 0.56).

**Table 1:** Average concentration of radon and thoron.

**Hospital**	**Radon level (Bq/m**^**3**^**)**	**Thoron level (Bq/m**^**3**^**)**
Imam Khomeini (ra)	6.8 ± 4.4	2.8 ± 2.8
Taleghani	11.6 ± 3.9	3.8 ± 2.75
Imam Reza (AS)	13.7 ± 4.3	4.64 ± 4.84

**Table 2:** Multiple regression model determining the influence of geological and meteorological parameters on indoor radon and thoron concentration.

Variable	On radon		On thoron	
	Coef.	[%95Conf. Interval]	Coef.	[%95Conf. Interval]
**Geographical location**				
Imam Khomeini (ra)	1		1	
Taleghani	−2.4	[−16.2 – 11.4]	-0.1	[−4.1 – 4]
Imam Reza (AS)	7.8	*[2 – 13.6]	4.3	[0 – 8.7]
**Soil type**				
clay with a little pasty	1		1	
clayey gravel with sand	4.2	*[1 - 7.36]	1.3	[−1.4 - 4]
Soil porosity	8.1	[−2.1 – 18.2]	2.9	[−5.3 – 11.1]
Indoor temprature	19	[−183.5 – 221.5]	44.2	*[30.3 – 58]
Outdoor temprature	−17.2	[−220.4 – 186]	−45.2	*[−59.1 – −31.4]
Indoor-Outdoor temperature differential	−17.7	−223 – 187.6]	−44.5	*[−58.6 – -30.5]
Indoor humidity	−0.4	[−7.3 – 6.6]	−1.5	*[−1.9 – -1]
Outdoor humidity	0.2	[−5 – 5.4]	0.5	*[0.2 – 0.9]
Indoor pressure	0.5	[−11.1 – 12]	0.5	[−0.3 – 1.3]
Outdoor pressure	−0.6	[−16 – 14.7]	−1.9	*[−2 – -0.8]
Windspeed	−0.7	[−3.3 – 1.8]	−1	*[−1.1 – -0.8]
Rainfall	−0.9	[−3.3 – 1.4]	−0.2	*[−0.3 – -0.02]

* Statistically significant.

The model indicated that the amount of radon in constructed buildings on soil with clayey gravel and sand feature, with a factor of 4.2 and a significant difference, was more than those constructed on soil with clay texture and little pasty. On the other hand the coefficient of thoron was minus (1.3) and a significant difference was not observed. Generally with increased the soil porosity, the indoor radon and thoron concentrations increased 8.1 and 2.9 times, respectively.

## 2 Experimental design, materials and methods

### 2.1 Description of study area

**Fig. 2:**
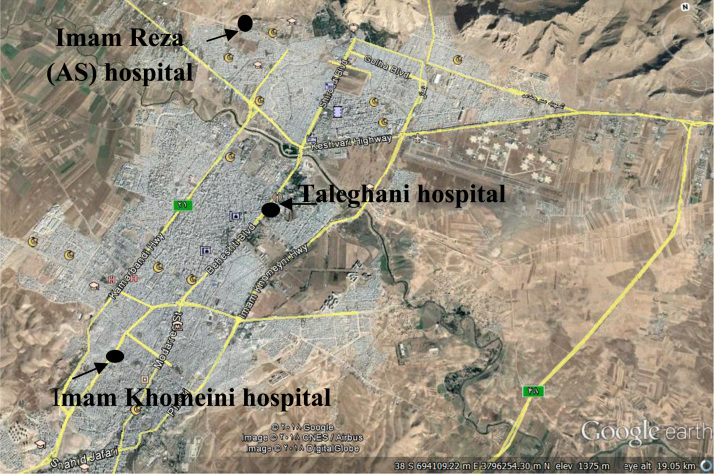
Location of the studied hospitals in Kermanshah city.

**Fig. 3:**
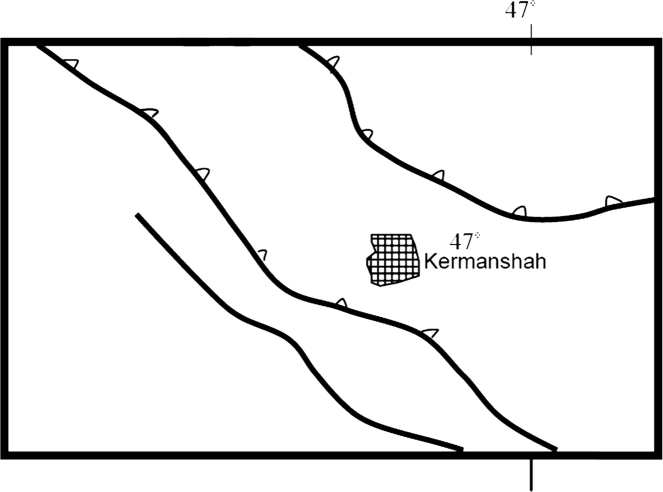
Faults surrounding Kermanshah city.

The radon and thoron concentrations were determined in three hospitals in Kermanshah, West part of Iran (Fig. 2). Kermanshah is located in 34 °18′51″N 47 °03′54″E with a cold semi-arid climate. The average temperature in summer and winter is 44.1 °C and −27 °C, respectively, and the average annual temperature is 14.3 °C. The geological formation of the area mainly consists of radiolarites type sedimentary rows. It has upper Triassic to Cretaceous rocks including sediments from deep areas that among them sedimentary rows of radiolarites and carbonates along with ophiolite rocks were in the highest level [11]. Kermanshah sited in the high Zagros area which is surrounded by faults from north to south (Fig. 3).

### 2.2 Measurement and data collection

The indoor radon and thoron concentration were measured using the RTM-1688-2 radon meter, from october to december 2012 which coincided with fall in Iran. 34 samples were determined in selected sites from different parts of three studied hospitals, three times (once per month). A total of 102 samples were collected for determine the indoor radon and thoron concentrations. In each measurement, after 150 min of continuous air suction, the radon and thoron concentrations simultaneously were reported with 95% confidence interval by the radon meter. Information about the type and porosity of the underlying soil collected from Soil Mechanics laboratory of Kermanshah. Meteorological parameters such as temperature, relative humidity, atmospheric pressure, rainfall and wind speed were obtained from Kermanshah Weather Bureau. Meanwhile, the radon meter (RTM-1688-2) is equipped with humidity, pressure and temperature sensors and in each measurement, these parameters were measured and reported by the device. All sampling and measuring of radon and thoron were performed according to the standard method [9], [11], [12], [13], [14].

### 2.3 Data analysis

Data obtained was analyzed using STATA 8. Univariate and multivariate linear regression model was used to determine the effects of geological and meteorological factors on indoor radon and thoron concentrations. In regression analysis, *P* < 0.05 was considered significant.
