# Supplementary material for: Data on the effect of geological and meteorological parameters on indoor radon and thoron level- case study: Kermanshah, Iran
Source: Data Brief. 2018 May 4;18:1945–51. doi: 10.1016/j.dib.2018.04.122 (PMC5998692; doi:10.1016/j.dib.2018.04.122)
Supplement: Supplementary file 1 — Supplementary material [file mmc1.doc]

None of the authors of this paper have any conflicts of interest to declare.
